# Baseline study for improving diagnostic stewardship at secondary health care facilities in Nigeria

**DOI:** 10.1186/s13756-022-01080-4

**Published:** 2022-05-03

**Authors:** Abiodun Egwuenu, Adaora Ejikeme, Sara Tomczyk, Anja von Laer, Olaniyi Ayobami, Oluwaseun Odebajo, Samuel Akhibi, Constance Agulanna, Osayande Osagie, Ugochi Stellamaris Inweregbu, Ridwan Yahaya, Tochi Okwor, Hannah Dada-Adegbola, Ikeoluwapo Ajayi, Abdulhakeem Olorukooba, Tim Eckmanns, Chinwe Lucia Ochu, Chikwe Ihekweazu

**Affiliations:** 1 Prevention Programmes and Knowledge Management, Nigeria Centre for Disease Control, Abuja, Federal Capital Territory Nigeria; 2 Nigeria Field Epidemiology Laboratory Training Program, Nigeria Centre for Disease Control, Abuja, Federal Capital Territory Nigeria; 3 Department of Infectious Disease Epidemiology, Robert Koch Institute, Berlin, Germany; 4 Medical Laboratory Unit, Maitama District Hospital, Abuja, Federal Capital Territory Nigeria; 5 Bwari General Hospital, Bwari, Federal Capital Territory Nigeria; 6 College of Medicine, University College Hospital, University of Ibadan, Ibadan, Oyo State Nigeria; 7 Department of Epidemiology and Medical Statistics, Faculty of Public Health, College of Medicine, University of Ibadan, Ibadan, Oyo State Nigeria; 8 Department of Community Medicine, Ahmadu Bello University, Zaria, Kaduna State Nigeria

**Keywords:** Sepsis, Blood culture, Antibiotic use, Diagnostic stewardship, Nigeria

## Abstract

**Background:**

Blood culture diagnostics are critical tools for sepsis management and antimicrobial resistance (AMR) surveillance. A baseline study was conducted to assess reported sepsis case finding, blood culture diagnostics, antimicrobial susceptibility testing (AST) and antimicrobial use at secondary health care facilities to inform the development of diagnostic stewardship improvement strategies in Nigeria.

**Methods:**

A cross-sectional online survey was conducted among 25 public secondary health care facilities in Abuja, Federal Capital Territory (FCT) and Lagos State in Nigeria to evaluate the capacity for pathogen identification and AST. Data were then prospectively extracted on all patients with reported suspected sepsis from electronic medical records from selected departments at two facilities in the Federal Capital Territory from October 2020 to May 2021 to further assess practices concerning sepsis case-finding, clinical examination findings, samples requested, and laboratory test results. Data were descriptively analysed, and a multivariate logistic regression analysis was conducted to determine factors associated with blood culture requests.

**Results:**

In the online survey, 32% (8/25) of facilities reported performing blood cultures. Only one had access to a clinical microbiologist, and 28% (7/25) and 4% (1/25) used standard bacterial organisms for quality control of media and quality control strains for AST, respectively. At the two facilities where data abstraction was performed, the incidence of suspected sepsis cases reported was 7.1% (2924/41066). A majority of these patients came from the paediatrics department and were outpatients, and the median age was two years. Most did not have vital signs and major foci of infection documented. Blood cultures were only requested for 2.7% (80/2924) of patients, of which twelve were positive for bacteria, mainly *Staphylococcus aureus*. No clinical breakpoints were used for AST. Inpatients (adjusted odds ratio [aOR]: 7.5, 95% CI: 4.6–12.3) and patients from the urban health care facility (aOR:16.9, 95% CI: 8.1–41.4) were significantly more likely to have a blood culture requested.

**Conclusion:**

Low blood culture utilisation remains a key challenge in Nigeria. This has implications for patient care, AMR surveillance and antibiotic use. Diagnostic stewardship strategies should focus on improving access to clinical microbiology expertise, practical guidance on sepsis case finding and improving blood culture utilisation and diagnostics.

## Background

In 2017, the Nigeria antimicrobial resistance (AMR) Technical Working Group identified limited utilisation of diagnostics and inadequate laboratory quality assurance as major factors affecting effective pathogen identification and antimicrobial susceptibility testing (AST) at health care facilities in Nigeria [1]. This contributes to the spread of AMR and undermines the ability to make effective patient management decisions. It also hinders the development of a functional AMR surveillance system [2].

Blood culture diagnostic is a critical tool for guiding clinical therapy decisions among patients with suspected bloodstream infections and sepsis. Sepsis is defined as a “life-threatening organ dysfunction caused by a dysregulated host response to infection” [3]. Sepsis is often caused by bacteria such as *Staphylococcus aureus, Streptococcus pyogenes, Klebsiella* spp*., Escherichia coli,* and *Pseudomonas aeruginosa* [3, 4]. Africa bears a disproportionately high burden of sepsis-related deaths, with sub-Saharan Africa and South-East Asia contributing 85% of 48.9 million sepsis cases and 84.8% of 11 million related deaths reported globally [5]. Approximately two million cases (a figure that is suspected to be grossly underestimated) of sepsis-related deaths occur in Africa [6]. Blood culture diagnostics in suspected sepsis are often underutilised in low-resource settings such as Nigeria due to lack of financing for consumables (e.g., patients are often unable to cover the costs of such diagnostics), limited microbiological capacity, and inadequate reporting of results for use. This is especially true at secondary level facilities where shortages of human resources, laboratory infrastructure, logistics and financial resources are more acute [7].

According to the World Health Organization, diagnostic stewardship can be defined as the “coordinated guidance and interventions to improve appropriate use of microbiological diagnostics to guide therapeutic decisions. It should promote appropriate, timely diagnostic testing, including specimen collection, and pathogen identification and accurate, timely reporting of results to guide patient treatment” [8]. The design and implementation of effective diagnostic stewardship strategies should be guided by a good understanding of the existing diagnostic capacities and practice.

We conducted a baseline study to assess reported sepsis case finding, the use of blood culture diagnostics, AST, and antimicrobial use at secondary health care facilities to inform the development of diagnostic stewardship improvement strategies in Nigeria.

## Methods

The baseline study was conducted in the context of an ongoing collaboration between the Nigeria Centre for Disease Control (NCDC) and the Robert Koch Institute (Berlin, Germany), aimed at improving diagnostic stewardship and expanding antimicrobial resistance (AMR) surveillance capacity, particularly at secondary healthcare facilities in Nigeria.

This study consisted of two stages. In the first stage, a cross-sectional online survey was conducted in 2019 to evaluate the capacity for pathogen identification and AST at public secondary health care facilities in Abuja, Federal Capital Territory (FCT) and Lagos State in Nigeria. Abuja, the capital city of Nigeria located in the centre of the country, has three public tertiary and 21 public secondary health care facilities [9], in addition to community pharmacies and patent medicine vendors (i.e. informal drug sellers with minimal or no training) where patients can directly buy antimicrobials [10, 11]. Lagos has two public tertiary and 44 public secondary health care facilities [9, 12]. The national AMR surveillance assessment checklist was sent to the head of the microbiology laboratory and medical directors at 65 public secondary health care facilities in Lagos and FCT via email and WhatsApp, and 25 hospitals completed the survey.

Among these, four health care facilities were selected (i.e. one in an urban area and one in a rural setting in each state) because of their capacity to conduct blood culture diagnostics and antimicrobial susceptibility testing. A structured on-site assessment of these facilities was conducted by a team of experts (i.e. two microbiologists, a medical doctor and an epidemiologist) to validate the survey results on laboratory staffing capacity, existing infrastructure including laboratory equipment, culture media production, information, communication and technology facilities, and a quality management system.

In the second stage from October 2020 to May 2021, existing practices related to reported sepsis case finding, pre-analytics, laboratory testing and post-analytics data were prospectively abstracted from the electronic medical record (EMR) systems at the two selected health care facilities in the Federal Capital Territory. The two facilities have 105 (urban) and 60(rural) inpatient beds respectively making a total of 165 beds, which reflects the average inpatient volume in many secondary health care facilities in Nigeria [13, 14]. These facilities regularly use computerised EMR systems. Although some forms and archiving remain paper-based, the EMR systems capture patients demographics, medical history and notes, medications, test results, clinical operations and costs of services [15].

Data from the EMR were extracted using a pretested tool with Open Data Kit (ODK) v1.30.1 software [16]. The tool was adapted from the WHO Proof-of-Principle (PoP) AMR Routine Diagnostics Surveillance Project Protocol [2]. Data was abstracted on all patients with a reported suspected bloodstream infection or sepsis diagnosis (as listed in the EMR by the clinician) [17] in the following departments: Emergency, Paediatrics, Neonatal, Obstetrics and Gynaecology, General Outpatient and Medical wards. The reported diagnosis terms used included “sepsis”, “septicaemia”, “septic shock”, “septic arthritis”, “neonatal sepsis”, “bacteraemia” and, patients without these diagnoses listed but got a blood culture request or test done. Among these patients, extracted variables included sociodemographics, clinical examination findings, antibiotic use, samples requested, laboratory test results and therapy changes (i.e. data collected in the pre-analytic, analytic, and post-analytic phases of the diagnostic pathway).

Data was extracted at 8:00 am the following day after patient consultation, 72 h after sample collection (for preliminary microscopy, culture and sensitivity results) and 10 days after sample collection (for final laboratory results), respectively. For patients whose results were not posted on the EMR, data were sourced from the laboratory registers. Daily data quality checks were conducted to assess and correct for missing and/or erroneous data. Six research assistants and two epidemiologist supervisors were trained on data collection methods/processes and extracted the data accordingly.

Data were cleaned using MS Excel 2019 and analysed using IBM SPSS Statistics 25 [18] and R Version 4.1.0 [19]. Categorical variables were summarized using frequencies and proportions and numeric variables as medians and interquartile ranges (IQR). For logistic regression analysis, the variables hospital type, age group in years, gender, health insurance status, patient type, antibiotic use pre-consult and after consult were included in the model. Antibiotic use was reported according to the AWaRe classification of antibiotics: Access (i.e. first- or second-line treatments for common infections), Watch (i.e. applied only to a limited group of well-defined syndromes), Reserve (i.e. applied as a last resort to treat multi- or extensively-drug resistant bacteria) as defined by the World Health Organization [20]. Univariate and multivariate logistic regression analyses were conducted to assess patient and hospital factors with adjusted odds ratio (aOR) and corresponding 95% confidence intervals (CI).

## Results

Twenty-five secondary health care facilities participated in the online survey during the first stage of the baseline study (Table 1). Among these, only four laboratories (16%) reported the use of an electronic database for laboratory data management. While all facilities reported having at least one laboratory scientist and a technician, only one had a pathologist or clinical microbiologist. Four (16%) laboratories reported at least one staff having had training on pathogen identification, AST and/or AMR data analysis in the preceding year. Although 80% (20/25) reported having standard operating procedures (SOPs) for quality management, only 28% (7/25) reported use of standard bacterial organisms for quality control of media and only 4% (1/25) reported reference quality control strains for AST (Table 1). Eight (32%) laboratories reported performing blood culture diagnostics with a median of ten blood samples processed per month and six laboratories regularly performed AST. However, only one laboratory used any form of guideline to interpret the result of the AST.

**Table 1:** Reported capacities and practices at participating secondary healthcare facilities in Abuja and Lagos, Nigeria, 2019

Capacities and practices	n (%) N = 25
**A) Laboratory infrastructure**	
i. Perform blood cultures	8 (32)
ii. Perform routine cultures of urine, wound, stool and/or cerebrospinal fluid	24 (96)
iii. Perform routine AST	22 (88)
iv. Regular supply of electricity including local back-up	11 (44)
v. Has a database and information system for laboratory data management	4 (16)
**B) Staff and training**	
i. Has at least one pathologist	1 (4)
ii. Has at least one laboratory scientist and technician	25 (100)
iii. Has at least a data clerk	8 (32)
iv. Trained laboratory staff in pathogen identification and/or AST in the preceding year	4 (16)
**C) Quality management**	
i. Has SOP for sample collection and processing	21 (84)
ii. Has SOPs/workflows for the preparation and quality control of media	20 (80)
iii. Use standard bacterial organisms for quality control of media	7 (28)
iv. Has SOPs/workflows for the identification of bacterial isolates	18 (72)
v. Use reference quality control strains for quality assurance of AST	4 (16)
vi. Performs regular external quality assurance	1 (4)

*SOP: Standard Operating Procedures, AST: Antimicrobial Susceptibility Testing

In the second stage of the baseline study, clinicians reported a suspsected sepsis diagnosis in 7.1% (2924/41066) of all patients at the two secondary health care facilities in Abuja, Nigeria from October 12, 2020, to May 15, 2021 (Table 2). Majority of these cases came from the paediatrics department 64.2% (1876/2924) and were outpatients 82.2% (2404/2924). More than half (1523/2924) were males, and the overall median age was two years (IQR: 6.2; Table 2).

**Table 2:** Characteristics of suspected-sepsis patients in two secondary healthcare facilities, Abuja, October 2020 to May 2021

Characteristics	n (%) N = 2924
**Hospital**	
Rural	1777 (60.8)
Urban	1147 (39.2)
**Age**	
≤ 5 years	2105 (72.0)
6–14 years	430 (14.7)
15–34 years	197 (6.7)
35–54	130 (4.4)
≥ 55 years	62 (2.1)
**Sex**	
Male	1523 (52.1)
Female	1401 (47.9)
**Place of residence (LGA)**	
Bwari	1858 (63.6)
Abuja Municipal Area Council (AMAC)	928 (31.7)
*Other	138 (4.7)
**Patient type**	
Outpatient	2404 (82.2)
Inpatient	418 (14.3)
Emergency	102 (3.5)
**Department**	
Paediatric	1876 (64.2)
General outpatient	477 (16.3)
Emergency	491 (16.8)
Neonatal	41 (1.4)
Female/male medical ward	23 (0.8)
Obstetrics and Gynaecology	16 (0.5)
**Temperature (°C)**	
< 36	28 (1.0)
36–38	1141 (39.0)
> 38	387 (13.2)
Unknown	1368 (46.8)
**Heart rate (beats/min)**	
≤ 90	312 (10.7)
> 90	139 (4.8)
Unknown	2473 (84.6)
**Respiratory rate (breaths/min)**	
≤ 20	53 (1.8)
> 20	198 (6.8)
Unknown	2673 (91.4)
**Systolic blood pressure (mmHg)**	
< 100	24 (1.0)
100–130	116 (4)
> 130	83 (2.8)
Unknown	2701 (92.4)
**Neurologic characteristics**	
Altered mental state	22 (1)
Conscious and alert	901 (30.8)
Not specified	1996 (68.3)
**Suspected focus of infection**	
Gastrointestinal	432 (14.8)
Respiratory tract infection	207 (7.1)
Skin or soft tissue	46 (1.6)
Bone or joint	25 (0.9)
Urinary tract	24 (0.8)
Wound or burn	24 (0.8)
Central nervous system	21 (0.7)
Genital	10 (0.3)
Cardiac	7 (0.2)
Other*	31 (1.1)
Not stated	2097 (71.7)
**Leukocyte count (mcL)**	
< 4000	97 (3.3)
4000–12,000	1135 (38.8)
> 12,000	357 (12.2)
Unknown	1335 (45.7)
**Neutrophils (mcL)**	
< 1500	25 (0.9)
1500–8000	4 (0.1)
> 8000	1560 (53.3)
Unknown	1335 (45.7)
**Malaria parasite test**	
Positive	705 (24.1)
Negative	811 (27.7)
Pending	565 (19.3)
Unknown	843 (28.8)

*Other includes Abaji, Gwagwalada, Kuje, Kwali, Outside Abuja and Unknown

** Other includes ear, throat, eye, left leg, neck, muscles, pelvis

Overall, 13.2% (387/2924) of patients with a reported suspected sepsis diagnosis had a temperature reading indicative of fever (>38°C) documented and a majority did not have heart rate 84.6% (2473/2924), respiratory rate 91.4% (2673/2924), or blood pressure 92.4% (2701/2924) measurements documented, respectively. Among 28.3% (827/2924) patients with a documented main focus of infection, gastrointestinal tract 14.8% (432/2924) and respiratory tract 7.1% (207/2924) were the most commonly recorded. A total of 54.4% (1591/2924) patients had a documented full blood count (FBC) including 3.3% (97/2924) with a leukocyte count of < 4000 mcL and 12.2% (357/2924) with a count of > 12,000 mcL and 24.1% (705/2924) were reported as having a positive malaria parasite result (Table 2).

**Fig. 1:**
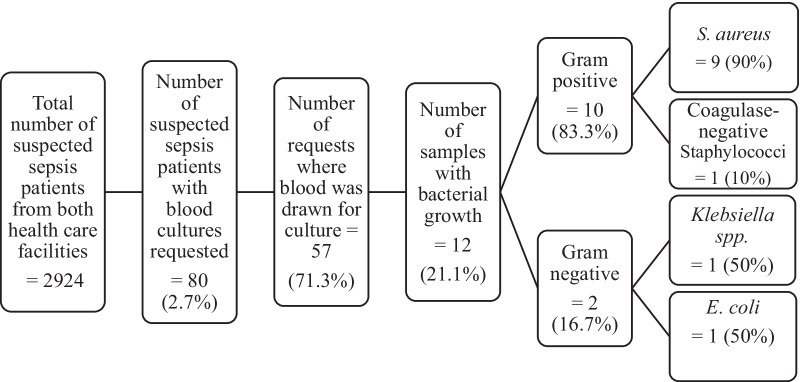
Blood-culture uptake by suspected-sepsis patients at two healthcare facilities, Abuja, October 2020 to May 2021

Out of 2,924 patients with a reported suspected sepsis diagnosis, blood culture was requested for 80 (2.7%) patients, and among these, 57 (71.3%) had a blood sample drawn for blood culture diagnostics. Twelve (21.1%) of the 57 samples were positive for bacteria with *Staphylococcus aureus* isolated from a majority of the cultures (Fig. 1). No clinical breakpoints were used for AST as sensitivity was reported only via visual inspection for a clearing around the antibiotic disc on the agar plate. Half of the *S. aureus* isolates were reported as resistant to erythromycin although, inconsistencies in antibiotic discs tested were found and no susceptibility or resistance to oxacillin was performed.

**Fig. 2:**
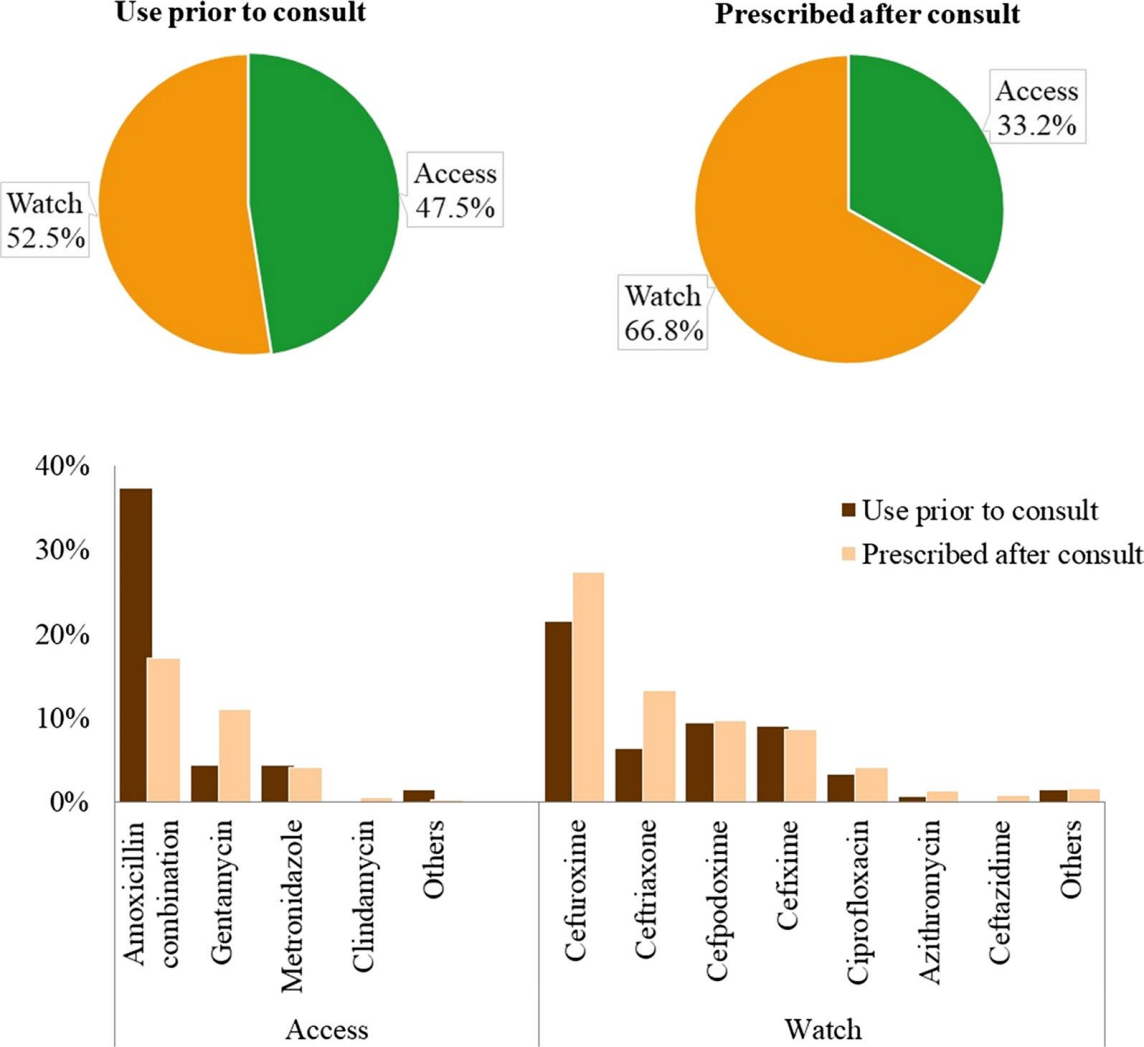
AWaRe classification of antibiotics pre and post hospital consultation in two secondary healthcare facilities Abuja. This includes N = 528 (antibiotics use prior to consultation) and N = 2436 (antibiotics prescribed after consultation)

Among patients with a reported sepsis diagnosis, the proportion on antibiotics increased from 15% (440/2924) pre-consultation to 67% (1958/2924) after consult, i.e. following contact with the doctor. After consultation, more antibiotics in the Watch category (66.8%, 1628/2436 antibiotic prescriptions) were prescribed for these patients in comparison to the Access category antibiotics (33.2%, 808/2436; Fig. 2), a increase from the proportion of Watch antibiotic used prior to consultation which was 52.5%. The commonest antibiotics used in the Access category were Amoxicillin combination, Gentamycin and Metronidazole, while Cefuroxime, Ceftriaxone and Cefpodoxime constituted the commonest antibiotics used from the Watch category (Fig. 2).

Patients with a reported suspected sepsis diagnosis at the urban healthcare facility were significantly more likely to have a blood culture requested than those at the rural healthcare facility (adjusted odds ratio [aOR]:16.9, 95% CI: 8.1–41.4). Furthermore, inpatients were significantly more likely to have a blood culture requested than emergency and outpatients (adjusted odds ratio [aOR]:7.5, 95% CI: 4.6–12.3) (Table 3). Factors such as health insurance status and age did not appear to play a significant role in the request for blood cultures.

**Table 3:** Patient- and hospital-level factors associated with blood culture request in two secondary healthcare facilities, Abuja

Variable	Blood culture request	Bivariate	Multivariate	
	Yes (%) N = 80	No (%) N = 2844	OR (95% CI)	aOR (95% C1)
**Hospital**				
Urban	73 (91.3)	1074 (37.8)	17.2 (7.9–37.5)	**16.9 (8.1–41.4)**
Rural	7 (8.8)	1770 (62.2)	Reference	Reference
**Age (years)**				
≤ 5	54 (67.5)	2051 (72.1)	0.3 (0.1–0.8)	0.7 (0.2–2.1)
6–14	8 (10.0)	422 (14.8)	0.2 (0.07–0.7)	0.7 (0.2–2.8)
15—34	7 (8.8)	190 (6.7)	0.4 (0.1–1.4)	0.5 (0.1—1.8)
35—54	6 (7.5)	124 (4.4)	0.6 (0.2–1.9)	0.6 (0.2—2.5
≥ 55	5 (6.3)	57 (2.0)	Reference	Reference
**Gender**				
Female	41 (51.3)	1360 (47.8)	1.1 (0.7–1.8)	–
Male	39 (48.8)	1484 (52.2)	Reference	-
**Health insurance status**				
Not enrolled	64 (80.0)	2131 (74.9)	1.3 (0.8–2.3)	–
Enrolled	16 (20.0)	713 (25.1)	Reference	–
**Patient type**				
Inpatients	42 (52.5)	376 (13.2)	7.6(4.7–12.0)	**7.5 (4.6–12.3)**
Emergency	3 (3.7)	99 (3.5)	2.1(0.6–6.8)	1.1 (0.2–3.7)
Outpatients	35 (43.8)	2369 (83.3)	Reference	Reference
**Patients on antibiotics before consultation**				
Yes	4 (5.0)	436 (15.3)	0.3 (0.1–0.8)	0.8(0.2–2.0)
No	76 (95.0)	2408 (84.8)	Reference	Reference
**Patients on antibiotics after consultation**				
Yes	49 (61.3)	1909 (67.1)	0.8 (0.5–1.2)	–
No	31 (38.8)	935 (32.9)	Reference	–

*Significant values are in bold

## Discussion

An assessment of 25 hospitals in Lagos and the Federal Capital Territory (former and present capital cities of Nigeria) showed that laboratory infrastructure remains weak, few laboratory staff reported recent training, use of SOPs or access to information technology tools remains minimal, and there is a dearth of pathologists and clinical microbiologists at secondary level health care facilities. The low incidence of suspected sepsis in two secondary health care facilities following a review of the EMR for records of the clinical judgement made by prescribers may be attributed to the meagre requests for blood culture. Laboratory diagnostics should guide patient management of health conditions, but limited access to and use of such services impedes accurate diagnosis and results in poorer health outcomes among patients. It also hinders availability of quality microbiologic data for AMR surveillance to inform empiric treatment guidelines and policy.

These findings highlight the urgent need for improved evidence-based interventions with participatory approaches to change practice particularly at secondary level health care facilities [21, 22]. These results should also be interpreted in the context of the Nigerian system. In Nigeria, primary health care facilities are governed by the local government area, secondary health care facilites by the States and teritary health care facilities by the Federal government. The secondary health care facility exists to “provide specialised services to patients referred from the primary health care level through out-patient and in-patient services at hospital centers for general, medical, surgical and paediatric patients” [23]. Users of secondary health care facilities often live within the same administrative area, which is different from tertiary hospitals where at least half of the clientele reside out of State, seeking highly specialised services and care at these facilities.

At the two health care facilities where data were abstracted over seven months, less than one in ten of patients in the department studied were found to have a reported suspected sepsis diagnosis, and this was largely among children 1–5 years in paediatrics and the outpatient department. This finding again should be interpreted in the context of the Nigerian health care system. There are less absolute number of inpatients in both hospitals, given more limited bed volume at secondary health care facilities, with 16 out of 17 patients within the hospital at any point in time being outpatients. Oftentimes, sepsis patients who are severely ill that present at these hospitals and require admission are referred to other secondary and tertiary health care facilities in the State, and these patients may not be captured on the EMR. In this largely outpatient setting, clinicians may also be using the term “suspected sepsis” as synonym for fever whose cause is yet to be identified and as a reason for prescribing antibiotics empirically which is otherwise known as providing “antibiotic cover” for suspected bacterial infections. This highlights the need for defined national algorithm that would guide stepwise management of febrile cases, provide a quality assurance system that ensures compliance with the set guidance and engender increased access to the required laboratory diagnostics [24]. In addition, a preponderance of sepsis among paediatric patients was demonstrated in this study similar to other studies in Nigeria and other low-resource settings [25–27]. This is also likely a result of poor access to vaccines and poor hygiene or infection prevention and control measures.

As it relates to sepsis case-finding practices, a majority of patients with a reported suspected sepsis diagnosis did not have their vital signs documented. This may be due to the lack of patient notes and quality data capture, but it could also be related to the choice of clinical criteria that doctors and nurses measure, record and use during clinical examinations at these hospitals. Evaluation of vital signs (i.e., body temperature, heart rate, respiratory rate, and blood pressure) is an important triage tool during clinical examination of both children and adults, for early recognition, diagnosis, and management of sepsis [28], particularly for patients admitted in the emergency setting (mainly in overloaded and most resource-limited ones). In low-resource settings, the systemic inflammatory response syndrome (SIRS) criteria (i.e., fever or hypothermia, tachycardia, tachypnea, leukocytosis, or leukopenia) and, more recently, the quick Sequential Organ Failure Assessment (qSOFA) criteria (i.e. increased respiratory rate, altered mentation, decreased systolic blood pressure) have often been cited as screening tools to identify patients with suspected sepsis. However, their usefulness in routine practice has been questioned given that the SIRS criteria have demonstrated high sensitivity but low specificity and the qSOFA was validated on patients with a suspected infection [7]. In a review of the best practices for blood cultures in low- and middle-income countries, Ombelet et al. suggest a revised set of clinical indications for blood culture sampling that could be more indicative of sepsis in such settings, which includes the presence of fever or hypothermia and one sign of severity such as, hypotension, confusion, increased respiratory rate, suspicion of severe localised infection, or suspicion of other severe infection [7].

Identification of the focus of infection is also important in sepsis management and optimisation of treatment, especially for cases where the site of infection can be removed or drained as seen in abdominal infections and soft-tissue abscesses. Although this study found the gastrointestinal and respiratory tracts to be common foci of infection among the suspected sepsis patients as seen in other Nigerian studies, majority of patients did not have foci of infection recorded, highlighting again the potential areas for improvement during clinical examination and data recording [24, 25]. In our study, 1 out of 5 suspected sepsis patients tested positive for malaria parasite. Malaria is a common cause of fever with significant morbidity and mortality in Nigeria [29–31] and fever is also a common symptom of sepsis. While people who contract malaria are at risk of developing sepsis and could potentially benefit from antibiotics especially in malaria-endemic regions and low resource settings like Nigeria [32], the evidence is conflicting. Guidance on diagnostic stewardship and sepsis case finding should also include malaria diagnosis using the national algorithm and early treatment while awaiting blood culture results.

Blood culture is an important diagnostic tool for pathogen identification to guide appropriate patient and sepsis management. Only 2.7% of all patients with a reported suspected sepsis diagnosis in this study were sent for blood culture, and only 1.9% had an actual blood sample drawn. This is lower than the findings from a previous study in Nigeria where about 12.5% of the patients who met sepsis criteria had a blood culture taken to guide therapy [33]. It differs even more significantly from findings in high-resource settings such as in the study by Otto et al. where more than 80% of patients were reported to have had blood sampling done for cultures [34]. The low rate of blood culture requests may be attributed to the fact that patients are expected to pay for blood culture diagnostics out-of-pocket which is often a financial burden that cannot be met, and clinicians may be wary to make this request due to the long turnaround time for blood cultures. According to this study's multivariate analysis, inpatients and those from the urban health care facility were significantly more likely to have a blood culture requested despite the fact that there were more suspected sepsis patients among outpatients compared to in-patients. Health insurance status was not associated with blood culture requests, suggesting that access remains an overall issue for all patients. Such poor utilisation of blood culture diagnostics has been shown to contribute to delayed patient recovery, missed diagnosis of sepsis resulting in delay in the institution of targeted antibiotics and long hospitalization [35]. Diagnostic stewardship improvement strategies should consider engagement with clinicians to increase the use of the laboratory, particularly in secondary level health care facilities; and the provision of essential commodities such as blood culture bottles, in order to improve access and availability of quality diagnostics.

Among the limited number of isolates, the most common bacteria from sepsis cases in this baseline study were *Staphylococcus aureus*, Coagulase-negative Staphylococci, *Klebsiella spp*. and *Escherichia coli*, a distribution similar to other studies in Nigeria [36, 37]. Overall, only one-fifth of blood culture samples in this study yielded growth. Low positive yield may be due to high contamination rates and quality issues along the pre-analytic and analytic pathway, e.g., inadequate asepsis during sample collection, sub-optimal transport and handling of samples, as seen in other large hospital studies [37]. These high rates of contamination along with long result turnaround time or lack of reporting causes mistrust in the diagnostic pathway and results. Accordingly, diagnostic stewardship strategies should include not only laboratory quality improvement efforts but also improved communication mechanisms to build trust between clinicians and laboratory scientists.

Low positive culture yields are also influenced by the use of antibiotics by patients before presenting to facilities [37]. In our baseline study, 3 out of 20 suspected sepsis patients reporting to the healthcare facilities were already on antibiotics pre-consult. This could indicate the antibiotic misuse and overuse i.e. procuring such prescription-only medicines over the counter [38]. After consultation, the proportion of patients on antibiotics increased to 14 out of 20 patients. In addition, two-thirds of the antibiotics used in our study were in the Watch category. This is in contrast with the target set by the World Health Organization for measuring appropriate, which is Access antibiotics should constitute 60% of all antibiotics consumed by 2023 [39, 40]. Increased use of Watch antibiotics and broad-spectrum or high-priority agents such as cephalosporins have been reported in Nigeria and other low-resource settings [41–44], while the overuse of Access antibiotics, often first and second choice therapy for common infections, has also been described in such settings [45, 46]. Such findings have important local implications for antimicrobial stewardship programs and prioritisation of antimicrobial stewardship interventions.

This baseline study is a large-scale study and arguably the first of its kind in Nigeria. The use of standard tools for data collection makes it reliable. However, there are limitations that should be considered. In the first stage, the online survey was completed by the heads of laboratories at 25 out of 65 public secondary health care facilities, so results may have been influenced by non-response bias. In the second stage, although the selected facilities reported regularly using EMR systems, the extraction of data on certain variables such as patients’ clinical characteristics were often incomplete. This was likely due to both lack of measurement during clinical examinations and poor data recording. Laboratory data were extracted from the EMR and manual laboratory registers, but this does not exclude the possibility of missing data and misclassification bias.

## Conclusion

The study provides important baseline information on the diagnostic process and antibiotic use among patients with suspected sepsis in secondary health care facilities in Nigeria, which will be used to inform diagnostic stewardship improvement strategies. Low blood culture utilisation remains a key challenge in these settings. Key study findings highlighted the need for improved access to clinical microbiology expertise particularly at the secondary health care facility level, renewed practical guidance on sepsis case finding, and antibiotic use.

## Data Availability

The data that support the findings of this study are available from the Nigeria Centre for Disease Control, Abuja, Nigeria but restrictions apply to the availability of these data, which were used under license for the current study, and so are not publicly available. Data are, however, available from the authors upon reasonable request and with permission from the Nigeria Centre for Disease Control.

## Abbreviations

AMR: Antimicrobial Resistance
AST: Antimicrobial Susceptibility Testing
BC: Blood culture
BSI: Bloodstream Infections
EMR: Electronic Medical Records
FBC: Full blood count
FCT: Federal Capital Territory
GLASS: Global Antimicrobial Resistance Surveillance System
ICU: Intensive Care Unit
LMIC: Low- and Middle-Income Countries
NCDC: Nigeria Centre for Disease Control
RKI: Robert Koch’s Institute, Germany
SOP: Standard Operating Procedure
WHO: World Health Organization
